# Nab-paclitaxel plus gemcitabine as first-line treatment for advanced pancreatic cancer: a systematic review and meta-analysis

**DOI:** 10.7150/jca.29898

**Published:** 2019-07-23

**Authors:** Yiyin Zhang, Jin Xu, Jie Hua, Jiang Liu, Chen Liang, Qingcai Meng, Quanxing Ni, Si Shi, Xianjun Yu

**Affiliations:** 1 Department of Pancreatic Surgery, Fudan University Shanghai Cancer Center, No. 270 Dong'An Road, Shanghai 200032, PR China; 2 Department of Oncology, Shanghai Medical College, Fudan University, No. 270 Dong'An Road, Shanghai 200032, PR China; 3 Shanghai Pancreatic Cancer Institute, No. 270 Dong'An Road, Shanghai 200032, PR China; 4 Pancreatic Cancer Institute, Fudan University, No. 270 Dong'An Road, Shanghai 200032, PR China

**Keywords:** nab-paclitaxel plus gemcitabine, advanced pancreatic cancer, meta-analysis.

## Abstract

To evaluate the effectiveness of nab-paclitaxel plus gemcitabine (NG) as a first-line treatment for advanced pancreatic cancer. A meta-analysis was performed to assess the impact on the objective response rate (ORR), survival rate and grade 3/4 adverse events. Of the 2,056 patients included from 26 studies, the median overall survival ranged from 6.9 months to 24.7 months, with a 1-year survival rate of 45.2% (95%CI: 35.8% -54.5%). The 6-month progression-free survival rate was 41.0% (95%CI: 30.5% - 51.4%), and the ORR was 31.6% (95%CI: 26.7% - 36.6%). Fifty locally advanced pancreatic cancer (LAPC) patients underwent surgery and had an R0 resection rate of 52.0%. No death was caused by toxicity, and 1,329 grade 3/4 adverse events were reported in 1,353 patients. NG has been proven to reduce tumours with an acceptable toxicity profile in metastatic pancreatic cancer. This analysis further demonstrates the efficacy and safety of NG for treating LAPC.

## Introduction

The prognosis for pancreatic cancer (PC) remains poor, and by 2030, PC will become the second leading cause of cancer-related deaths in the United States 1. Only approximately 20% of patients are suitable for resection when first diagnosed; the remaining 80% are diagnosed with locally advanced pancreatic cancer (LAPC) or metastatic pancreatic cancer (MPC). LAPC is deemed unresectable because direct operation might leave positive resection margins, which jeopardize overall survival (OS) to a degree similar to that in cases not involving resection 2. Radical surgical resection remains the only curative treatment for PC, and it is used in LAPC patients after primary treatment; however, randomized controlled trials (RCTs) studying systemic chemotherapy have been performed in this patient group. Nab-paclitaxel, an albumin-bound nanoparticle form of paclitaxel, has demonstrated significant clinical benefit over gemcitabine monotherapy for MPC by improving the intratumoural concentration of gemcitabine 3. The efficacy and safety of nab-paclitaxel plus gemcitabine (NG) was validated in the MPACT study, which showed response rates of 23% and 35% survival at one year 4. Several studies on the use of NG to treat advanced pancreatic cancer (APC) have emerged in recent years. Therefore, we performed a meta-analysis to evaluate NG as a first-line treatment for APC patients.

## Materials and methods

This study was performed according to the procedures of Preferred Reporting Items for Systematic Reviews and Meta-Analyses statement 5. The primary outcome measure was the objective response rate (ORR), which was defined as the time from the initial NG treatment until a change in disease status. Secondary outcome measures were disease control rate (DCR); 1-year and 2-year survival rates; grade 3/4 adverse events; post-NG surgical resection rate and R0 resection rate.

### Search strategy and study selection

We searched for all eligible studies in PMC, PubMed, Embase, MEDLINE and Web of Science from inception to November 28, 2018. The regular NG regimen consisted of nab-paclitaxel (100 - 125 mg/m^2^) and gemcitabine (1000 mg/m^2^) on days 1, 8, and 15 every 28 days. Search terms were defined as (Abraxane OR nab-paclitaxel OR albumin-bound paclitaxel) AND gemcitabine AND pancreatic. The search results were limited to human studies in English only. Conference abstracts were included because some clinical trials related to APC have not yet been published as papers. The inclusion criteria were as follows: (1) eligible patients diagnosed with APC; (2) patients did not receive surgery, but adjuvant chemo(radiation)therapy was allowed if received at least 6 months prior to NG treatment; (3) NG was accepted as first-line treatment without any other concurrent chemo(radiation)therapy; (4) non-clinical research, including reviews, meta-analysis, case reports, systematic reviews, basic experiments and letters to editors, was excluded.

### Data extraction and quality assessment

Two independent reviewers (Yiyin Zhang and Jie Hua) designed the search strategy and assessed abstract eligibility. Disagreements were settled by discussion, and a consensus was achieved. Information extracted from related studies included the following: title, first author's name, number of eligible patients, tumour stage, ORR, DCR, progression-free survival (PFS), 6-month PFS rate, OS, 1-year and 2-year survival rates, surgical conversion rate and R0 rate. If progression or survival data were not provided in the text but only in graphs and figures, Engauge Digitizer version 4.1 (http://digitizer.source-forge.net/) was used to extract numerical values.

To assess the quality of the included studies with full text, the Newcastle-Ottawa Scale (NOS) was applied by 2 independent reviewers. RCTs were evaluated according to their JADAD score for the following: randomization, double blinding, withdrawals and dropouts. Scores ranged from 0 to 5, and ≥ 3 was considered high-quality literature 6. Non-RCTs were evaluated separately and judged primarily on 3 parameters: selection of the study groups, comparability of the groups and outcomes of cohort studies. A maximum of 9 points was assigned to each study, and scores of 5 to 9 were considered high quality 7.

### Statistical analysis

All of the analyses were performed using the STATA SE 12.0 package (StataCorp, College Station, Texas, USA) and Comprehensive Meta-analysis 2.0 (Biostat, New Jersey, USA). We used the random-effects model if I^2^ > 50% or *P* < 0.1; otherwise, the fixed-effects model was applied. The results were considered statistically significant at *P* < 0.05. Heterogeneity was determined by χ² tests and I^2^ statistics as described by Higgins and Thompson. Subgroup analysis was performed according to LAPC and MPC diagnosis; NG and FOLFIRINOX (FFX) treatment for MPC; and the efficacy of NG and non-NG treatment in MPC.

## Results

### Literature search and study characteristics

**Figure 1:**
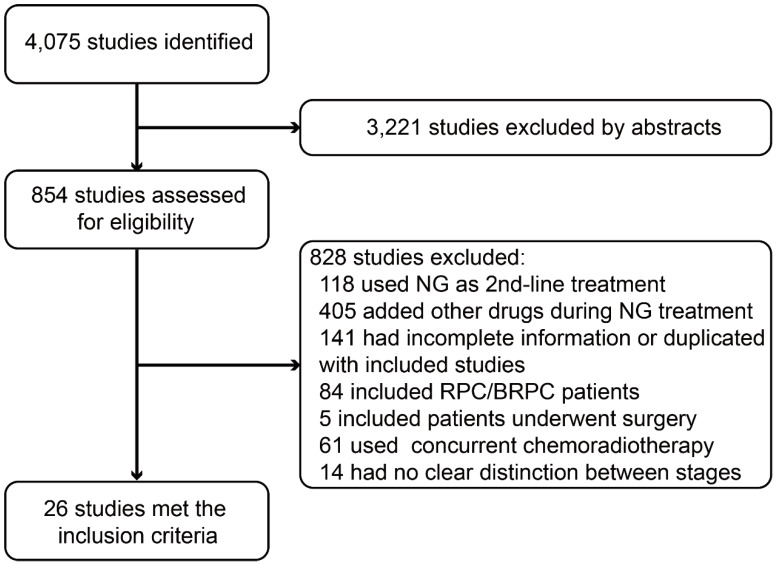
Flowchart of the search process of our study. Abbreviations: NG=nab-paclitaxel plus gemcitabine; RPC=resectable pancreatic cancer; BRPC=borderline resectable pancreatic cancer.

In PMC, PubMed, Embase, MEDLINE and Web of Science, we identified 4,075 studies, among which 3,221 were excluded based on their abstract, and 854 were potentially appropriate. We screened the remaining 854 studies and excluded 828 studies for the reasons listed in Figure 1. Thus, we included 26 articles examining a total of 2,056 PC patients in this systematic review and meta-analysis 8-33. Fifteen studies were retrospective, and 11 studies were prospective. Among all included patients, 210 had LAPC, and 1,846 had MPC. The characteristics of all of the included studies meeting the inclusion criteria for this meta-analysis are provided in Table 1 and Table 2.

### Primary endpoint

**Figure 2:**
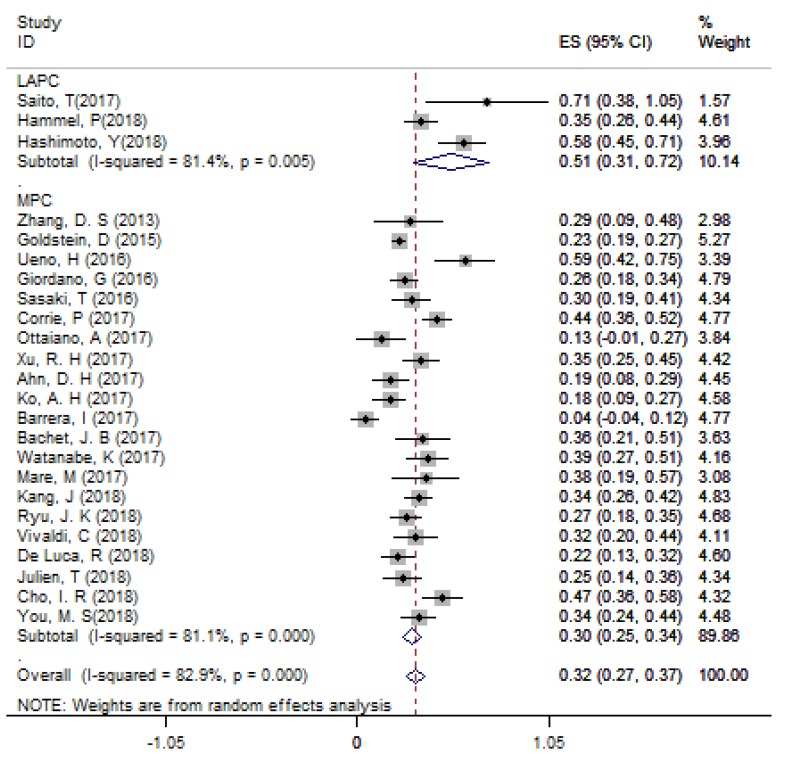
The overall ORR for APC. Abbreviations: ORR=objective response rate; APC=advanced pancreatic cancer.

According to single arm analysis, the overall ORR in 24 accessible studies was 31.6% (95%CI: 26.7% - 36.6%) for APC. In the subgroup analysis, the ORR was 51.3% (95% CI: 30.6% - 71.9%) in 3 LAPC studies and 29.5% (95% CI: 24.7% - 34.4%) in 21 MPC studies (Figure 2). MPC patients in the non-NG group had an ORR of 25.3% (95%CI: 13.7% - 37.0%). Four studies compared the efficacy of NG and FFX as first-line treatments for MPC patients, and the ORR of the FFX group was 33.2% (95% CI: 14.3% - 52.1%), which was lower than the 37.7% ORR (95% CI: 32.2% - 43.3%) in the NG group.

### Secondary endpoints

**Figure 3:**
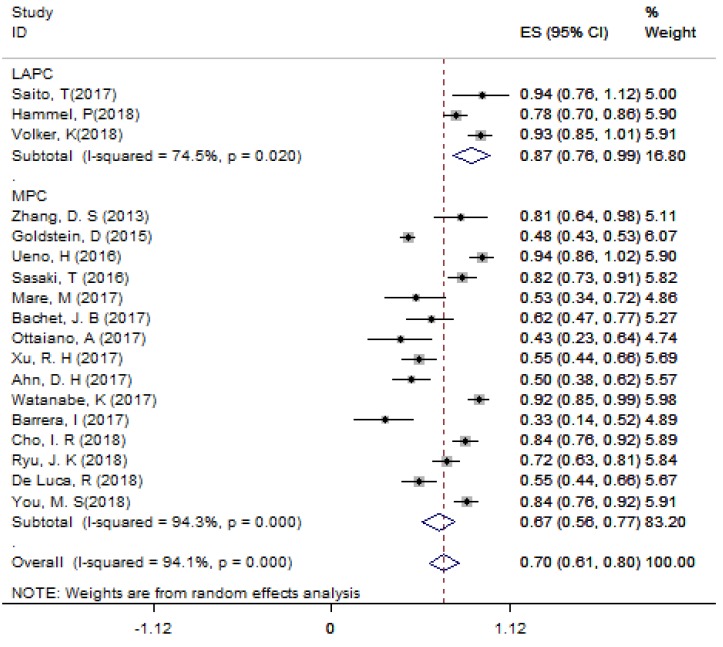
The DCR for LAPC and MPC. Abbreviations: DCR=disease control rate; LAPC=locally advanced pancreatic cancer; MPC=metastatic pancreatic cancer.

**Figure 4:**
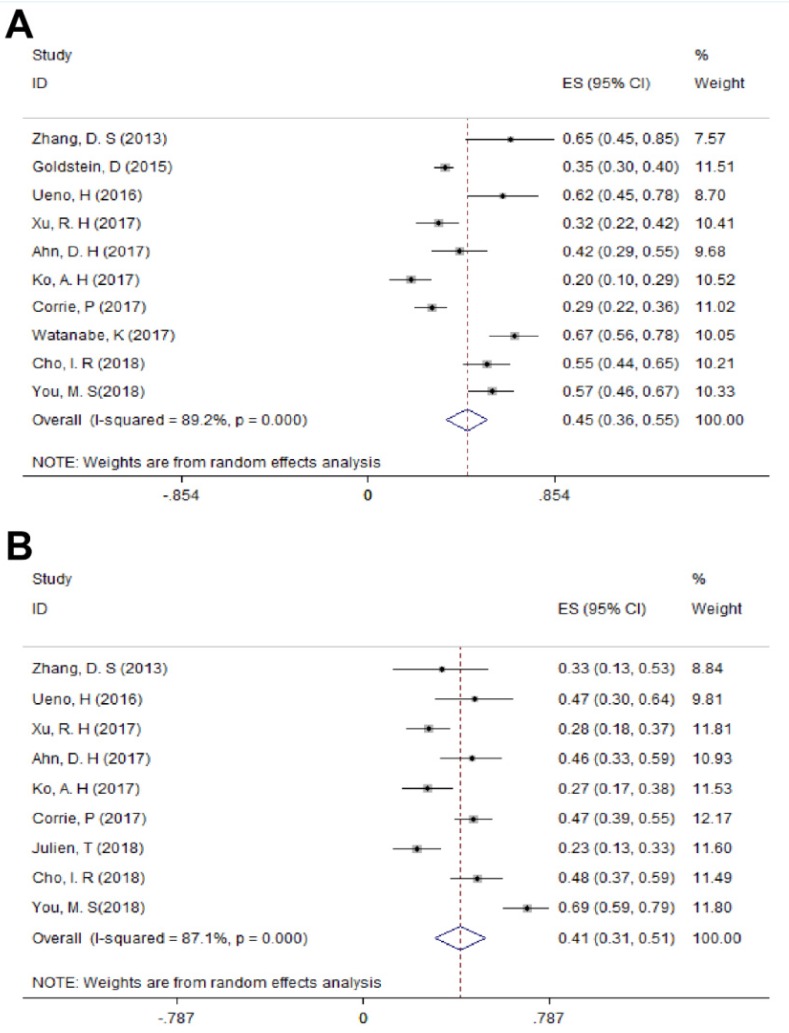
The 1-year survival rate A) and 6-month PFS rate B) in MPC. Abbreviations: PFS=progression-free survival; MPC=metastatic pancreatic cancer.

For LAPC, the DCR generated from 3 studies was 87.4% (95%CI: 75.8% - 98.9%; Figure 3). Fifty (24.6%) patients underwent surgical resection after 4 cycles of NG administration, and the R0 rate was 52.0% (26/50), ranging from 44.0% to 100.0%. For MPC, the DCR generated from 15 studies was 66.7% (95%CI: 56.4% - 77.1%; Figure 3); the median OS ranged from 6.9 months to 14.7 months across 19 studies. The 1-year survival rate evaluated for 10 studies was 45.2% (95%CI: 35.8% -54.5%; Figure 4A), the 2-year survival rate evaluated for 3 studies was 6.6% (95%CI: 0.1% -13.1%). The number of studies evaluating the 2-year survival rate decreased because most studies terminated before 24 months. The median PFS ranged from 4.0 months to 8.4 months across 18 studies, and the 6-month PFS rate was 41.0% (95%CI: 30.5% - 51.4%; Figure 4B) for 9 studies. In the non-NG group, the DCR for 3 studies was 42.0% (95%CI: 7.8% - 76.3%), and the 1-year survival rate was 28.6% (95%CI: 16.5% - 40.7%).

Twenty-one studies used the same NG dose as the MPACT trial, except 1 study including 57 patients who used a modification of the NG dose described as gemcitabine (1000 mg/m^2^) and nab-paclitaxel (125 mg/m^2^) on days 1 and 15 of every 28 days 30. The median OS of this study was 10.0 months (ranging from 5.9 to 13.0 months), and the median PFS was 5.4 months (ranging from 4.1 to 7.4 months) 15. One study changed only the dose of nab-paclitaxel to 120 mg/m^2^. The median number of NG-administered cycles ranged from 2 to 9 8. The FFX regimen was as described in the PRODIGE 4 trial, which was a 2-h intravenous infusion of oxaliplatin (85 mg/m^2^) followed by a 2-h intravenous infusion of leucovorin (400 mg/m^2^), and irinotecan was given 30 minutes later (180 mg/m^2^) via 90-minute intravenous infusion. Fluorouracil was given immediately after via intravenous bolus at a dose of 400 mg/m^2^ followed by a continuous intravenous infusion of 2400 mg/m^2^ over a 46-hour period biweekly 34. The modified usage of FFX was not elaborated in the methods of the included studies.

For LAPC, 113 patients in 2 studies were analysed for grade 3/4 adverse events. Two studies used a full dose of NG as described in the MPACT trial, and 76 grade 3/4 adverse events were reported. For MPC, 1,183 patients in 14 studies received the full NG dose that the MPACT trial prescribed, and 833 haematological (Table 3) and 386 non-haematological grade 3/4 adverse events (Table 4) were reported, with neutropenia and leukopenia being the most common adverse events. One study used a modified NG dose (on days 1 and 15 every 28 days), and 33 grade 3/4 adverse events were reported 15. One study that used an NG dose of 120 mg/m^2^ reported 5 grade 3/4 adverse events. Eight studies did not report toxicity data. No deaths were attributed to NG treatment 8. Four hundred and twenty-four (65.1%) grade 3/4 adverse events were reported among 651 MPC patients treated with a non-NG regimen.

### Quality assessment

The results of the NOS scale and JADAD score showed that the quality of the included studies ranged from moderate to high (Table 5).

## Discussions

Numerous studies have focused on the efficacy and safety of NG for APC patients, although no definitive conclusions have been identified to date. This is the first meta-analysis summarizing the use of NG as a first-line APC treatment. Although the combination of nab-paclitaxel and gemcitabine was a milestone in MPC treatment, there remains a paucity of data regarding the use of NG to treat APC.

In 2013, Von Hoff DD and colleagues reported the first open-label randomized phase III study that included 431 MPC patients treated with nab-paclitaxel followed by gemcitabine, which achieved a median OS of 8.5 months (95% CI: 7.5 - 9.5 months) compared with 6.7 months (95% CI: 6.0 - 7.2 months) for those treated with gemcitabine alone. The 1-year survival rate exceeded 30% and reached 35%, significantly outperforming the 22% rate reported for gemcitabine monotherapy (GEM) (*P* < 0.001). The rates of peripheral neuropathy and myelosuppression were increased but acceptable and reversible. Since then, an increasing number of case reports and clinical studies assessing NG treatment for APC have been published. MPACT is the largest study exploring the efficacy and side effects in the MPC population thus far. Other studies with a smaller population (n = 1415) further confirmed the effectiveness and safety of NG, with a median OS ranging from 6.9 to 14.7 months and a 6-month PFS rate of 41.0%. Before the advent of NG, FFX was verified as an active APC regimen and exhibited a median OS of 4.3 months longer than GEM. However, the FFX study excluded patients older than 75 years and patients with an Eastern Cooperative Oncology Group status of 2, which might influence the results because performance status was an independent predictor of survival 4. Ventriglia J *et al* suggested that NG was effective and safe in an unselected population of elderly patients based on an evaluation of 46 patients with a median age older than 73 years 35. New studies directly comparing FFX and NG reported an ORR of 33.2% in the FFX group and 37.7% in the NG group. Kang J *et al* found that patients had a longer median OS in the NG group than in the FFX group (11.4 vs 9.6 months; *P* = 0.002), especially patients older than 65 years; these patients also had a higher Charlson score and peritoneal metastasis 36. NG also caused less grade 3/4 neutropenia, diarrhoea and peripheral neuropathy incidences than modified FFX treatment 29. Although NG caused considerable peripheral neuropathy incidence, patients with grade 3/4 recovered to grade 1 or lower in a median of 29 days, which was more rapid than patients with cumulative oxaliplatin toxicity 4. Moreover, the development of peripheral neuropathy was associated with an improved median OS (grade 3 vs grade 0: 14.9 vs 5.9 months, HR= 0.33; *P* < 0.0001). The result was inspiring for clinical physicians, as it provided reliable evidence for choosing NG as first-line chemotherapy for patients with underlying medical conditions. The patients included in the non-NG group in our analysis showed a grade 3/4 toxicity rate of 65.1%, mainly because some data for non-haematological events were not able to be extracted. The difference in CA19-9 levels should also be considered when selecting treatments for MPC patients because those with an abnormal CA19-9 level had better outcomes with gemcitabine than with FFX 34. In the exploratory MPACT study, CA19-9 levels were reduced significantly more in the NG group than in the gemcitabine-alone group, and the CA19-9 decrease might help identify patients with a survival benefit by week eight 37. The prognosis was worse for patients with normal CA19-9 levels than for those with decreased CA19-9 levels, indicating that the level of CA19-9 reduction might screen out patients insensitive to NG. This spares NG-insensitive patients from unnecessary side effects, preserving their strength for other treatments and improving their quality of life (QoL). A recently published study used propensity-matched analysis to compare the effectiveness of FFX and NG in the neoadjuvant setting for resectable and borderline resectable PC. The reduction of pN1 disease and improvement of 4.9-month OS in the FFX group compared with the NG group indicated that more attention should be paid to the selection of specific patient groups that would actually benefit from either FFX or NG treatment 38. Attempts have been made to improve the efficacy and reduce the side effects of sequential therapy. The PRODIGE 37-FIRGEMAX trial provided a 2-month alternate MPACT regimen and 2-month FOLFIRI regimen for patients with a performance status (PS) of 0-2. The results showed that peripheral neuropathy decreased 35% compared to that found for the MPACT regimen, and a 60% 6-month PFS rate was achieved. FIRGEMAX appeared to be feasible and effective for MPC with a tolerable toxicity profile and guaranteed treatment for more than 2 months 17.

Patients with LAPC rarely underwent surgical resection because the 2016 ASCO guidelines recommended that treatment for LAPC should focus on local control and QoL, and no clear evidence supported one regimen over another due to the lack of RCTs comparing different regimens in this specific population 39. However, Georgios G *et al* retrospectively included 415 LAPC patients who received FFX- and gemcitabine-based chemotherapy, and the results showed that patients who underwent resection (20%) after 5 months of neoadjuvant chemotherapy had a median OS of 35.3 months (similar to patients with resectable disease) compared with 16.2 months for those who progressed after neoadjuvant chemotherapy. Additionally, patients who received N0 and R0 resection lived significantly longer than those who received N1-2 and R1 resection. However, no significant difference between neoadjuvant treatments was observed in that study 40, indicating that the efficacy of neoadjuvant chemotherapy could help predict the prognosis of LAPC patients. In the studies that we pooled for this analysis, 24.6% of LAPC patients completed surgical conversion after NG with an R0 rate of 52.0%. In 2 studies comprising 113 patients, 76 (67%) grade 3/4 adverse events were reported. These results were similar to those in LAPC patients who received FFX but had a lower R0 rate (24.6% vs 28%; 52.0% vs 74%; 67% vs 60%) 41. RCTs are required to compare the efficacy and safety of FFX and NG for treating LAPC. The current ongoing phase II LAPACT included 107 LAPC patients treated with NG, and preliminary results indicated that CA19-9 levels decreased by ≥ 50% in 75.3% patients with a median time to failure of 8.8 months, which was 2.2 months longer than expected. The LAPACT trial intended to recruit 220 patients to evaluate NG as an optimal first-line plan to improve the surgical conversion rate in LAPC 12.

The role that chemoradiotherapy plays in APC is ambiguous. The LAP 07 trial showed that LAPC patients who received continued chemotherapy after controlled disease exhibited a longer median survival than those who proceeded to chemoradiotherapy 42. However, a recent study based on 13,004 LAPC patients in the National Cancer Database demonstrated that chemoradiotherapy was associated with a superior OS compared with chemotherapy alone (HR = 0.79; 95% CI: 0.76 - 0.83; *P* < 0.001) 43. Yamada S *et al* designed a chemoradiotherapy protocol using NG in combination with 50.4 Gy, which had a 50% (6/12) surgical conversion rate and a 100% 1-year survival rate with only one patient experiencing grade 4 toxicity 44. Stereotactic body radiotherapy (SBRT) has feasible, safe and effective properties when used to treat unresectable patients with comorbidities that preclude surgery, intensive chemotherapy and conventional radiation therapy 45. These recommendations were also supported by a large retrospective study of 8,450 LAPC patients who showed a survival advantage for SBRT treatment compared with CRT (HR = 0.84, *P* < 0.001) 46. Future randomized trials should consider SBRT for continuing treatment after NG for LAPC patients because no treatment can be substituted for surgical resection; improving the surgical conversion rate is of utmost importance.

Grade 3/4 adverse events pooled from prospective studies were higher than those from retrospective studies, indicating that the pooled adverse event rate might be more accurate in prospective studies. The modified NG dose resulted in nearly half the grade 3/4 adverse event rate as the full dose with similar median OS and PFS rates, thus providing a reliable treatment option for those with a poor PS. The 6-month QoL for the gemcitabine treatment arm was two times higher than that for the FFX arm (66% vs 31%, respectively; *P* < 0.001), in accordance with a recently published study showing that NG was associated with relatively tolerable toxicities, especially for MPC, which generally has a poorer PS and convenient free genetic testing, compared with FFX 47.

Nevertheless, several limitations should be mentioned. First, individual information was not presented in some of the included studies, which might contain unknown prognostic factors, combinations of other medications and additional comorbidities. Second, not all of the included studies were randomized clinical trials, leading to potential bias. Third, the PFS might be biased because only prospective studies used standardized imaging to evaluate treatment efficacy; the retrospective studies included in this analysis did not provide similar imaging.

The findings in this analysis may benefit a substantial proportion of APC patients, as LAPC and MPC account for most PC cases. The surgical conversion rate and R0 resection rate after NG treatment were considerable in LAPC. NG showed mild side effects in MPC patients compared with FFX. However, NG efficacy in treating LAPC is not conclusive due to the insufficient sample sizes of the included trials. Therefore, more high-quality RCTs with larger sample sizes are required to reveal true NG efficacy.

## Figures and Tables

**Table 1:** Characteristics of studies included in the meta-analysis.

Study	Year	No. of patients	Stage	ORR	DCR	Median OS (months)	95% CI	1-year survival rate	Median PFS (months)	95% CI	6-month PFS rate
**LAPC**											
Saito	2017	7	III	0.71	0.94	13.30	11.30 - 15.30	0.86	-	-	-
Hammel	2018	106	III	0.35	0.78	-	-	-	10.20	-	-
Volker	2018	42	III	-	0.93	-	-	-	-	-	-
Hashimoto	2018	55	III	0.58	-	24.70	15.50 - NR	-	-	-	-
**MPC**											
Zhang	2013	21	IV	0.29	0.81	12.17	9.49 - 14.84	0.65	4.43	4.01 - 4.83	0.33
Goldstein	2015	431	IV	0.23	0.48	8.70	7.89 - 9.69	0.35	5.50	4.50 - 5.90	-
Giordano	2016	118	IV	0.26	-	11.00	9.58 - 12.41	-	7.00	5.96 - 8.03	-
Sasaki	2016	70	IV	0.30	0.82	10.40	-	-	5.90	-	-
Ueno	2016	34	IV	0.59	0.94	13.50	10.60 - NR	0.62	6.50	5.10 - 8.30	0.47
Corrie	2017	146	IV	0.44	-	10.10	-	0.29	5.80	-	0.47
Mare	2017	26	IV	0.38	0.53	9.00	2.00 - 18.00	-	6.00	1.00 - 12.00	-
Bachet	2015	39	IV	0.36	0.62	9.20	6.00 - 13.60	-	4.90	2.10 - 7.70	-
Ottaiano	2017	23	IV	0.13	0.44	-	-	-	-	-	-
Xu	2017	83	IV	0.35	0.55	9.30	-	0.32	5.50	5.29 - 7.16	0.28
Ahn	2017	57	IV	0.19	-	10.00	5.90 - 13.00	0.42	5.40	4.10 - 7.40	0.46
Barrera	2017	24	IV	0.04	0.33	-	-	-	-	-	-
Ko	2017	66	IV	0.18	0.50	6.90	-	0.20	6.80	-	0.27
Watanabe	2017	65	IV	0.34	0.92	14.00	12.20 - NR	0.67	6.50	6.10 - 7.90	-
Cho	2018	81	IV	0.47	0.84	12.10	10.70 - NR	0.55	8.40	5.00- 11.80	0.48
Julien	2018	62	IV	0.25	-	-	-	-	-	-	0.23
Vivaldi	2018	57	IV	0.32	-	10.60	-	-	6.20	-	-
Ryu	2018	101	IV	0.27	0.72	14.70	-	-	7.30	-	-
Kang	2018	149	IV	0.34	-	11.40	-	-	6.80	-	-
Pacheco-Barcia	2018	25	IV	-	-	14.00	-	-	8.00	-	-
De Luca	2018	80	IV	0.23	0.55	8.00	7.13 - 8.86	-	5.00	3.86 - 6.13	-
You	2018	88	IV	0.34	0.84	14.20	11.80 - 20.30	0.57	8.40	7.10 - 13.20	0.69

Abbreviations: ORR=objective overall survival; Median OS=median overall survival; CI=confidence interval; Median PFS=median progression-free survival; NR=not reached.

**Table 2:** Characteristics of studies included in the non-NG group.

Study	Regimen	No. of patients	ORR	DCR	Median OS	1-year survival rate	Median PFS	6-month PFS rate
Goldstein	Gemcitabine	430	0.07	0.35	6.60	0.22	3.70	-
Ko	NG + apatorsen	66	0.18	-	5.30	0.22	2.70	0.27
Barrera	FFX	10	0.00	0.10	-	-	-	-
Bachet	N + simplified leucovorin and fluorouracil	75	0.35	-	11.60	-	6.40	-
Vivaldi	mFFX	81	0.36	-	11.50	-	6.40	-
Pacheco-Barcia	mFFX	21	-	-	14.00	-	8.00	-
Kang	FFX	159	0.34	-	9.60	-	5.10	-
Watanabe	mFFX	70	0.29	0.79	11.50	0.44	5.70	-
Julien	FIRGEMAX	65	0.40	-	15.80	-	-	0.45

Abbreviations: ORR=objective response rate; DCR=disease control rate; Median OS=median overall survival; Median PFS=median progression-free survival; NG=nab-paclitaxel plus gemcitabine; FFX=FOLFIRINOX; N=nab-paclitaxel; mFFX=modified FOLFIRINOX; FIRGEMAX=nab-paclitaxel plus gemcitabine and FOLFIRI.

**Table 3:** Grade 3/4 haematological toxicities.

Haematological toxicities	Study (number of events)	Total events
Leukopenia	Saito (3), Goldstein (127), Ueno (19), Xu (28)	177
Anemia	Hammel (12), Goldstein (56), Ueno (5), Cho (12), Xu (12), Ahn (8), Bachet (5), Ryu (23), You (19)	152
Thrombocytopenia	Goldstein (52), Ottaiano (2), Ueno (5), Zhang (1), Cho (5), Xu (8), Ahn (1), Bachet (7), Mare (4), You (5)	90
Neutropenia	Hammel (45), Goldstein (153), Ottaiano (5), Ueno (24), Zhang (2), Cho (38), Xu (27), Ahn (11), Bachet (12), Ryu (41), Pacheco-Barcia (3), Watanabe (29), Julien (31), You (34)	455
Lymphocytopenia	Ueno (5), De Luca (11)	16
Febrile neutropenia	Ueno (2), Zhang (2), Ahn (1), De Luca (10), You (5)	20

**Table 4:** Grade 3/4 non-haematological adverse events.

Non-haematological toxicities	Study (number of events)	Total events
Liver dysfunction	Ueno (1)	1
Stomatitis	De Luca (8), Bachet (7)	15
Neuropathy	Hammel (4), Goldstein (71), Ottaiano (7), Ueno (4), Zhang (1), Cho (15), Ahn (1), Bachet (4), Ryu (15), Watanabe (3), Mare (1), Julien (20), You (16)	162
Diarrhea	Goldstein (24), Ueno (2), Watanabe (1), Julien (2), You (1)	30
Appetite loss	Ueno (1)	1
Rash	Ueno (1), Julien (14)	15
Bilateral cellulitis	Saito (1)	1
Fatigue	Hammel (11), Goldstein (73), Ottaiano (4), Xu (11), Ahn (4), De Luca (27), Mare (2), Julien (2)	134
Nausea and vomit	Ueno (1), Cho (16), Ahn (1), You (3)	21
ALP abnormality	Ottaiano (3), Bachet (5)	8
Hyperglycemia	Ottaiano (2)	2
Hyponatremia	Ueno (2)	2
Hyperbilirubinemia	Ottaiano (3)	3

**Table 5:** Assessment of study quality.

**Newcastle-Ottawa scale for non-randomized controlled studies**					
**Study**	**Selection (0-4)**	**Comparability (0-2)**	**Outcome (0-3)**		**Total**
Saito	3	1	2		6
Zhang	3	1	2		6
De Luca	2	1	2		5
Barrera	3	1	3		7
Vivaldi	3	1	2		6
Ryu	2	1	2		5
Pacheco-Barcia	4	1	2		7
Watanabe	4	1	2		7
Mare	2	1	2		5
Giordano	3	1	2		6
Sasaki	2	1	2		5
Kang	3	1	2		6
Hashimoto	2	1	2		5
Ottaiano	3	1	2		6
Xu	3	1	2		6
Ahn	3	1	2		6
Cho	3	2	1		6
You	2	2	1		5
**JADAD score for randomized controlled studies**					
**Study**	**Randomization (0-2)**	**Double blinding (0-2)**	**Withdrawals and dropouts (0-1)**	**Total**	
Corrie	1	1	1	3	
Hammel	1	1	1	3	
Julien	2	1	1	4	
Bachet	2	2	1	5	
Volker	2	1	1	4	
Ueno	1	1	1	3	
Ko	2	2	1	5	
Goldstein	2	2	1	5	
					

## Abbreviations

PC: pancreatic cancer
LAPC: locally advanced pancreatic cancer
MPC: metastatic pancreatic cancer
OS: overall survival
RCTs: randomized controlled trials
NG: nab-paclitaxel plus gemcitabine
APC: advanced pancreatic cancer
ORR: objective response rate
DCR: disease control rate
GEM: gemcitabine
FFX: FOLFIRINOX
QoL: quality of life
SBRT: stereotactic body radiotherapy
PS: performance status
NOS: Newcastle-Ottawa scale
